# Phenotypic Diversity in Multiple Sclerosis Can Be Represented by Four Additive Symptom Modules

**DOI:** 10.3390/brainsci16070753

**Published:** 2026-07-16

**Authors:** Daniel B. Hier, Pavankumar Y. Srinivasula, Michael D. Carrithers

**Affiliations:** 1 Department of Neurology and Rehabilitation, College of Medicine, University of Illinois Chicago, Chicago, IL 60612, USA; mcar1@uic.edu; 2 College of Liberal Arts and Sciences, University of Illinois Chicago, Chicago, IL 60607, USA; psrin4@uic.edu

**Keywords:** multiple sclerosis, non-negative matrix factorization, phenotype, symptom modules, electronic health records, natural language processing, clinical heterogeneity

## Abstract

**Background:** Multiple sclerosis (MS) lacks a single invariant phenotypic core. Patients accumulate heterogeneous combinations of sensory, motor, cognitive, and autonomic impairments over time, reflecting lesions that are disseminated in time and space. Standard scales such as the Expanded Disability Status Scale (EDSS) distribute disability across functional systems, but do not explicitly represent MS phenotype as a mixture of latent symptom modules. **Methods:** We analyzed 4617 de-identified neurology progress notes from 577 patients with MS at a single academic medical center. A large language model (GPT-5.2) categorized each note with respect to 17 non-mutually-exclusive neurological phenotype features, and note-level features were aggregated to patient-level binary vectors. Non-negative matrix factorization (NMF) was applied to generate three-, four-, and five-module solutions. For each rank, we computed approximate variance captured, relative reconstruction error, and module-level feature loadings. In the preferred four-module solution, we derived patient-level module percentages, identified highly dominant (≥55%) and archetypal (≥70%) module profiles, and quantified admixture using Shannon entropy and the effective number of modules. **Results:** Three-, four-, and five-module NMF solutions showed similar approximate variance captured (52.7–54.3%) and reconstruction error (0.47–0.53), but the four-module solution provided the clearest clinical interpretation. The four latent modules were sensory-visual-pain, ataxic-spastic-falls, cognitive-psychologic-fatigue, and autonomic-bladder-bowel, aligning closely with established functional systems in MS. Most patients exhibited admixed phenotypes, with module entropies ranging from 0 (single-module dominance) to 1.386 (equal mixture) and effective modules spanning approximately 1 to 4. Using pre-specified thresholds, 154 patients (26.6%) were highly dominant in a single module and 72 (12.5%) were archetypal; these purer phenotypes were most often in the sensory-visual-pain module. **Conclusions:** MS phenotypic diversity in routine clinical practice can be parsimoniously represented as mixtures of four latent symptom modules rather than as positions along a single severity axis. Most patients show substantial admixture of sensory, motor, cognitive, and autonomic involvement, but a minority exhibit relatively pure or strongly dominant module patterns. This modular representation provides an interpretable framework for quantifying MS phenotype and for generating testable hypotheses about MS subtypes whose biological relevance remains to be established.

## 1. Introduction

Low-rank approximation methods, including non-negative matrix factorization, have been widely used for unsupervised EHR-based phenotyping because they can transform high-dimensional clinical data into transparent and interpretable patient- and feature-level factors [1]. Many neurological diseases have a readily recognizable phenotypic core that is shared across most cases. Alzheimer’s disease is typically anchored by a core of memory impairment, Parkinson’s disease is generally defined by a core of bradykinesia, rigidity, and resting tremor, and Charcot–Marie–Tooth disease usually presents with a core of sensory loss, hyporeflexia, weakness, and muscle atrophy. Multiple sclerosis (MS) differs in an important way: it lacks a single invariant phenotypic core. Some patients present with sensory symptoms, others with weakness, others with optic neuritis and visual loss, and others with ataxia or incoordination [2,3]. No single symptom complex defines all cases at onset. Traditional classifications by disease course (relapsing–remitting, secondary progressive, primary progressive) capture temporal pattern but do not fully describe the cross-sectional distribution of symptoms across functional systems.

The marked clinical heterogeneity of neurological disorders has also encouraged the development of artificial intelligence and machine learning approaches for disease diagnosis and phenotype characterization. Recent studies have applied gradient boosting for dementia detection [4], deep convolutional neural networks such as EfficientNet for brain tumor classification [5], and multivariate classification techniques for identifying MS phenotypes from neuroimaging and spectroscopic data [6], demonstrating the growing role of computational methods in neurological disease characterization.

Disease progression also differs from that of many other neurological disorders. In diseases such as Alzheimer’s disease and Parkinson’s disease, progression typically consists of gradual worsening of the same core symptoms that define the disorder at onset. As Parkinson’s disease advances, bradykinesia and rigidity generally worsen; as Alzheimer’s disease progresses, memory impairment deepens. In MS, by contrast, progression is often more complex. In some patients, initial symptoms worsen over time; in others, deterioration occurs in a stepwise manner, with new episodes adding deficits in domains that were not previously affected. Thus, clinical worsening in MS may reflect not only increasing severity within an existing symptom domain, but also expansion of symptoms into new domains.

As MS evolves, some patients remain relatively restricted to one or two domains of impairment, whereas others accumulate deficits across multiple domains over time [7,8]. These domains do not necessarily emerge simultaneously at onset, but may be recruited sequentially as the disease unfolds. This pattern suggests that MS progression reflects the additive recruitment of multiple symptom modules rather than progressive worsening of a single invariant symptom core. Although relatively “pure” phenotype profiles may be observed in MS (for example, predominantly motor or predominantly sensory syndromes), most patients can be viewed as reflecting an admixture of phenotype features that vary in both severity and domain.

Kurtzke recognized that the clinical picture of MS was modular, and he explicitly designed the Functional System Scores (FSS) to rate relatively independent symptom systems (pyramidal, cerebellar, brainstem, sensory, visual, etc.) that occur in varying admixtures within individual patients. Subsequent correlation analyses have confirmed that these systems are only weakly to moderately correlated, supporting their interpretation as partially independent axes of impairment rather than indicators of a single unidimensional disability construct [9,10]. Although Hobart and colleagues [11] correctly argue that the FSS should not be summed to yield a psychometric “total disability” score, the pattern of low–moderate intercorrelations is precisely what one would expect if MS phenotype is composed of separable symptom modules—making such multi-axis representations naturally amenable to decomposition by non-negative matrix factorization. In this respect, the FSS resemble profile scores such as verbal and performance IQ: their partial independence argues against a single monolithic “disability quotient” but supports a modular, multi-axis representation of function.

Non-negative matrix factorization (NMF) is a linear multivariate method that decomposes a non-negative data matrix *X* into the product of two lower-rank non-negative matrices *W* and *H*, such that *X* ≈ WH [12,13,14,15]. The columns of *W* represent latent modules, and the rows of *H* specify the contribution of each observed feature to those modules. Because all entries are constrained to be non-negative, NMF represents each observation as an additive combination of modules rather than as a mixture of opposing positive and negative loadings. In practical terms, NMF reduces a larger set of observed features to a smaller number of additive modules that summarize recurrent patterns in the data.

In early exploratory analysis of high-dimensional patient phenotype data, an important goal is to obtain a low-dimensional representation that supports both visualization and interpretation, ideally providing clinically meaningful axes and allowing each patient to be assigned a concise summary profile. Traditional unsupervised workflows often cluster patients in a high-dimensional feature space and then visualize them with nonlinear embedding methods such as t-SNE or UMAP; although these approaches can reveal regional structure, the resulting axes are not directly interpretable and the clinical meaning of cluster membership may be difficult to recover. In contrast, NMF directly reduces a larger set of correlated symptom features to a smaller number of additive modules and represents each patient as a weighted mixture of those modules. This additive constraint distinguishes NMF from methods such as principal component analysis (PCA) or independent component analysis (ICA), which allow negative loadings and thus represent data as combinations of opposing components.

In neurological disease, this additive structure is not merely a mathematical convenience but may reflect underlying biological reality: in MS, clinical deficits accumulate over time as lesions affect distinct motor, sensory, autonomic, and cognitive systems, so the observed phenotype can be viewed as a superposition of deficits across multiple functional domains rather than a single severity axis. Under this interpretation, NMF provides a natural framework for modeling MS phenotype as a superposition of independent functional system axes; each patient is represented as a weighted combination of latent factors corresponding to these domains, rather than as a point along a single severity dimension. We therefore asked whether the heterogeneity of MS phenotype, as documented in routine clinical notes, could be summarized by a small number of additive symptom modules and whether individual patients could be represented as relatively pure or admixed combinations of those modules. Given the complexity and diversity of neurological signs and symptoms in MS, this framework suggests that categorization of MS patients within this framework might yield three patterns: (1) a small number of patients with nearly pure presentations of just one symptom module (e.g., archetypal motor or sensory presentations), (2) many more patients with presentations suggesting dominance of just one or two symptom modules (e.g., predominantly motor–sensory, etc.), and (3) the bulk of patients with highly admixed phenotypes drawing from multiple symptoms modules (e.g., mixed motor–sensory–cognitive–autonomic presentations).

The purpose of this approach was not simply to reduce 17 phenotype features to four modules, but to create a quantitative phenotype space in which each patient can be described by both a predominant clinical pattern and an effective number of contributing modules.

## 2. Methods

### 2.1. Neurology Notes

A total of 12,661 de-identified neurology clinical notes were obtained from a REDCap database at the University of Illinois Hospital, the primary teaching hospital of UI Health, for the period from 14 January 2016, through 2 September 2022.

Notes were eligible for inclusion if associated with a diagnosis of multiple sclerosis (ICD-10-CM G35) and classified as Progress Note encounters; patient-directed Visit Summaries were excluded. Notes were further screened with GPT-5.2 to retain full neurology physician notes; an initial 600-word-length threshold, introduced to exclude very short telephone-call summaries and administrative notes lacking sufficient narrative for reliable phenotyping, became redundant once note-type screening was applied, as inclusion was determined by note content rather than note length and the cohort is therefore not biased toward verbose clinicians.

After deduplication, 4617 notes remained for analysis. Use of de-identified clinical documentation for research was approved by the Institutional Review Board of the University of Illinois (Protocol No. 2017-0520Z).

The 577-patient cohort had a mean age of 46.5 years (SD 12.7; range 19–80); 437 (75.7%) were female and 140 (24.3%) male. By race, 287 (49.7%) were Black/African American, 132 (22.9%) White, 128 (22.2%) of other or multiracial background, 6 (1.0%) Asian, and 24 (4.2%) not reported; 108 (18.7%) identified as Hispanic/Latino and 444 (76.9%) as non-Hispanic.

Each note represented a narrative summary of a patient encounter and served as the unit of analysis. Prior to annotation, notes were converted to JavaScript Object Notation (JSON) format, with each note represented as a single JSON object. The primary data field in each object was note_text, which contained the full note in plain-text format. Associated metadata included note_id, patient_id (de-identified), age, sex, race, note_length (characters), num_notes (number of notes available for that patient), note_date, note_type (e.g., Progress Note), clinic (e.g., Neurology), and elapsed_days (days from the first note to the current note) (see Table A1).

### 2.2. Categorization of Neurology Notes by Phenotype

Each neurology note was categorized with respect to 17 non–mutually exclusive neurological phenotype features, including weakness, sensory symptoms, pain, ataxia, and cognitive impairment. Phenotyping was performed using the OpenAI API and GPT-5.2, following previously described methods in which GPT-5.2 achieved human-level performance against clinician-annotated ground truth (F1 = 0.80, recall/sensitivity = 0.92, precision = 0.73) [16]. GPT-5.2 assigned each of the 17 categories as present or absent at the note level via a structured JSON prompt and returned a short free-text justification for each assignment, restricted to findings documented as present in the encounter; historical, absent, or uncertain findings were not coded as present. To confirm correct handling of negation and temporality, we reviewed the discordant cases between GPT-5.2 and the two human annotators in the companion validation study; the discordances did not suggest systematic confusion of negated findings (e.g., “no ataxia”) with present findings, or of current with historical findings (e.g., “blurred vision” versus “history of blurred vision”). Per-feature (phenotype-level) precision and recall for all 17 categories are reported in that companion study; GPT-5.2 exhibited a sensitive-but-inclusive profile (higher recall, more false positives than the human annotators), which is relevant to interpreting module composition.

Because most patients contributed multiple notes over time, note-level phenotype assignments were aggregated at the patient level. For each patient, the full set of notes was summarized as a 17-dimensional binary phenotype burden vector, where 1 indicated that a phenotype feature was present in at least one note and 0 indicated that the feature was absent from all notes. Repeated mention of the same phenotype across multiple notes did not increase the score. After aggregation, patients with no phenotype features were excluded, leaving 577 patients for non-negative matrix factorization. The final phenotype burden vector represented cumulative burden across all available notes, with each dimension constrained to binary values (0 or 1). The decision to aggregate across all available notes, rather than rely on a single encounter, was motivated by the long-recognized variability and incompleteness of physician documentation [17,18,19]: documentation practices differ across physicians and encounter types, and clinicians economize on documentation effort rather than re-recording every known finding at every visit. Cumulative aggregation across all available notes was therefore adopted to compensate for encounter-level documentation gaps. Patients contributed a median of 4 notes (range 1–64).

### 2.3. Non-Negative Matrix Factorization

Non-negative matrix factorization (NMF) was performed using the NMF implementation in scikit-learn (v1.7.2; sklearn.decomposition), with max_iter = 5000, random_state = 0, and init = nndsvd. We examined 2-, 3-, 4-, and 5-module solutions by varying n_components.

For each candidate rank, model fit was summarized using relative reconstruction error, computed as the Frobenius norm of the residual matrix divided by the Frobenius norm of the original data matrix:

(1) $$\frac{{∥X-WH∥}_{F}}{{∥X∥}_{F}}$$

Lower relative reconstruction error was interpreted as indicating better reconstruction of the original phenotype matrix. Candidate solutions were also evaluated for interpretability, stability, and parsimony of the resulting phenotype modules.

**Figure 1:**
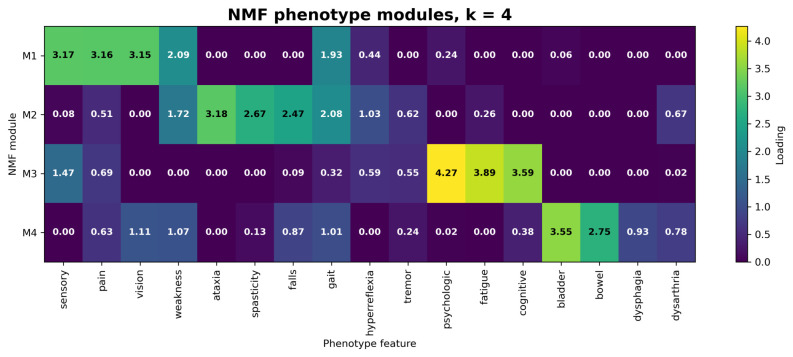
Heatmap of feature loadings for the preferred 4-module NMF solution with sensory-visual-pain, ataxia-spastic-falls, cognitive-psychologic-fatigue, and autonomic-bladder-bowel as primary phenotypes of each module. Note that weakness and gait load significantly on modules M1 and M2.

Using the highest-loading feature as a provisional label, heat maps were constructed for the 3-, 4-, and 5-module solutions (Figure 1, Figure A2 and Figure A3).

Although the 4-module solution was intermediate with respect to approximate variance explained and reconstruction error, it showed the greatest clinical interpretability and was therefore selected as the preferred solution. The four modules were named “sensory-visual-pain”, “ataxic-spastic-falls”, “cognitive-psychologic-fatigue”, and “autonomic-bladder-bowel” on the basis of their feature-loading patterns (Table 1). We use the term “module” throughout to refer to these NMF-derived additive components, reserving “subtype” for disease-course classifications (relapsing–remitting, secondary progressive, primary progressive); the term “cluster” is avoided, as patients are represented as additive mixtures across modules rather than assigned to discrete groups.

Selection of the number of NMF components is a recognized challenge in unsupervised EHR phenotyping; prior work has emphasized that model order should be chosen by balancing reconstruction error, stability, interpretability, and clinical validity rather than by fit alone [1].

To assess reproducibility in the absence of an external cohort, we performed repeated internal split-sample validation. In each of 100 random 80/20 patient splits, the four-module NMF was fit on the training 80% and the held-out 20% was projected onto the fixed training-set loadings. Because NMF components are unordered, modules were matched to their full-cohort counterparts using one-to-one maximum-correlation assignment based on the Pearson correlation of the 17-dimensional feature-loading vectors. The four modules reproduced with mean matched loading correlations of 0.984, 0.995, 0.979, and 0.982 (median 0.987–0.997); the top three features of each module were preserved in 83–100% of splits; held-out reconstruction error (0.472) was comparable to the full-cohort error (0.466); and held-out dominant-module assignments agreed with full-cohort assignments in 86% of patients (median 0.88).

Solution stability was further assessed with 100 repeated 90% subsampling runs, in each of which a random 90% sample of patients was drawn without replacement and the four-module model was refit using the same matching procedure. Matched loading correlations were summarized by their median, interquartile range, minimum, and maximum (Table A2); subsampling yielded median matched loading correlations of 0.994–0.998 (feature-loading interquartile ranges 0.16–0.22). Ten random initializations produced a matched loading correlation of 1.000 for all modules, indicating an essentially unique solution.

### 2.4. Computed Features

Using the feature loadings of the four-module model (Figure 1), the contribution of each module to the total patient-level loading was calculated and normalized to sum to 100%. For each patient, we identified the dominant module as the module with the highest percentage loading. We also identified patients with a strongly dominant module, defined as one module accounting for more than 55% of the total loading, and near-archetypal patients, defined as those in whom one module accounted for more than 70% of the total loading (Table 2). Finally, empirical archetypal patients were identified as vertices of the convex hull of the normalized four-dimensional NMF loading space using scipy.spatial.ConvexHull (scipy v1.15.2), which is based on the Qhull algorithm.

Entropy was calculated from the row-normalized NMF weights, with each patient’s four module weights scaled to sum to 100%. The effective number of modules was then computed as $e^{H}$, where *H* is Shannon entropy.

### 2.5. Plotting and Visualization

**Figure A1:**
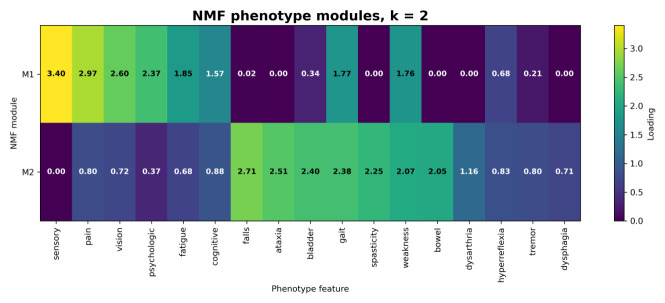
Heatmap of feature loadings for the 2-module NMF solution. Numeric values represent feature loadings. This lower-rank solution provides a coarse separation of phenotype features but does not preserve the clinically interpretable four-domain structure selected for the main analysis.

**Figure A2:**
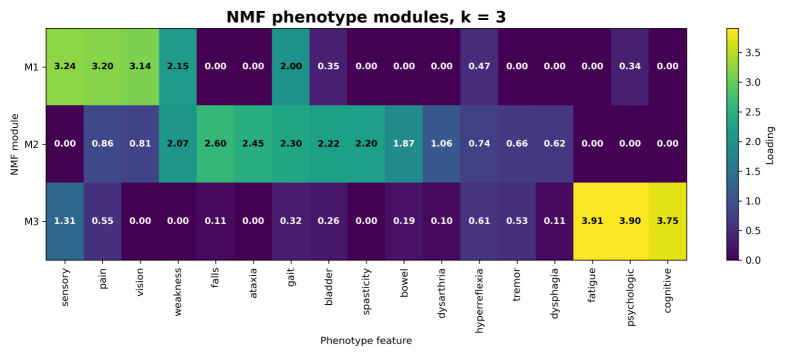
Heatmap of feature loadings for the 3-module NMF solution. This solution partially separates sensory-visual-pain, motor-gait, and cognitive-fatigue features, but combines several clinically distinct domains that are better separated in the 4-module solution.

**Figure A3:**
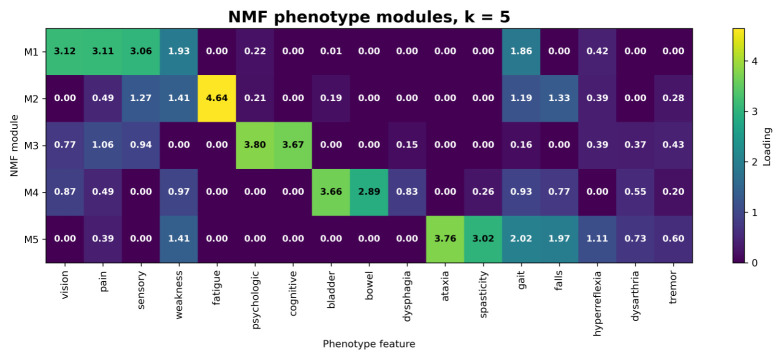
Heatmap of feature loadings for the 5-module NMF solution. This higher-rank solution further separates selected features, including fatigue, but provides less parsimonious clinical interpretation than the preferred 4-module solution.

Heat maps showing feature loadings for the 2-, 3-, 4-, and 5-module solutions were created using the seaborn library (v0.13.2) (Figure 1 and Figure A1, Figure A2 and Figure A3). Because the four-module solution was selected as the preferred NMF model, patients could be represented in a tetrahedral space in which the four module weights summed to 100%. This constraint made symmetric tetrahedral visualization possible. The tetrahedron plot was created with Matplotlib (v3.10.9), using mpl_toolkits.mplot3d.

To visualize individual triangular faces of the tetrahedron as simplex plots, we used the python-ternary library in combination with Matplotlib. Histograms were created with Matplotlib.

## 3. Results

**Figure 2:**
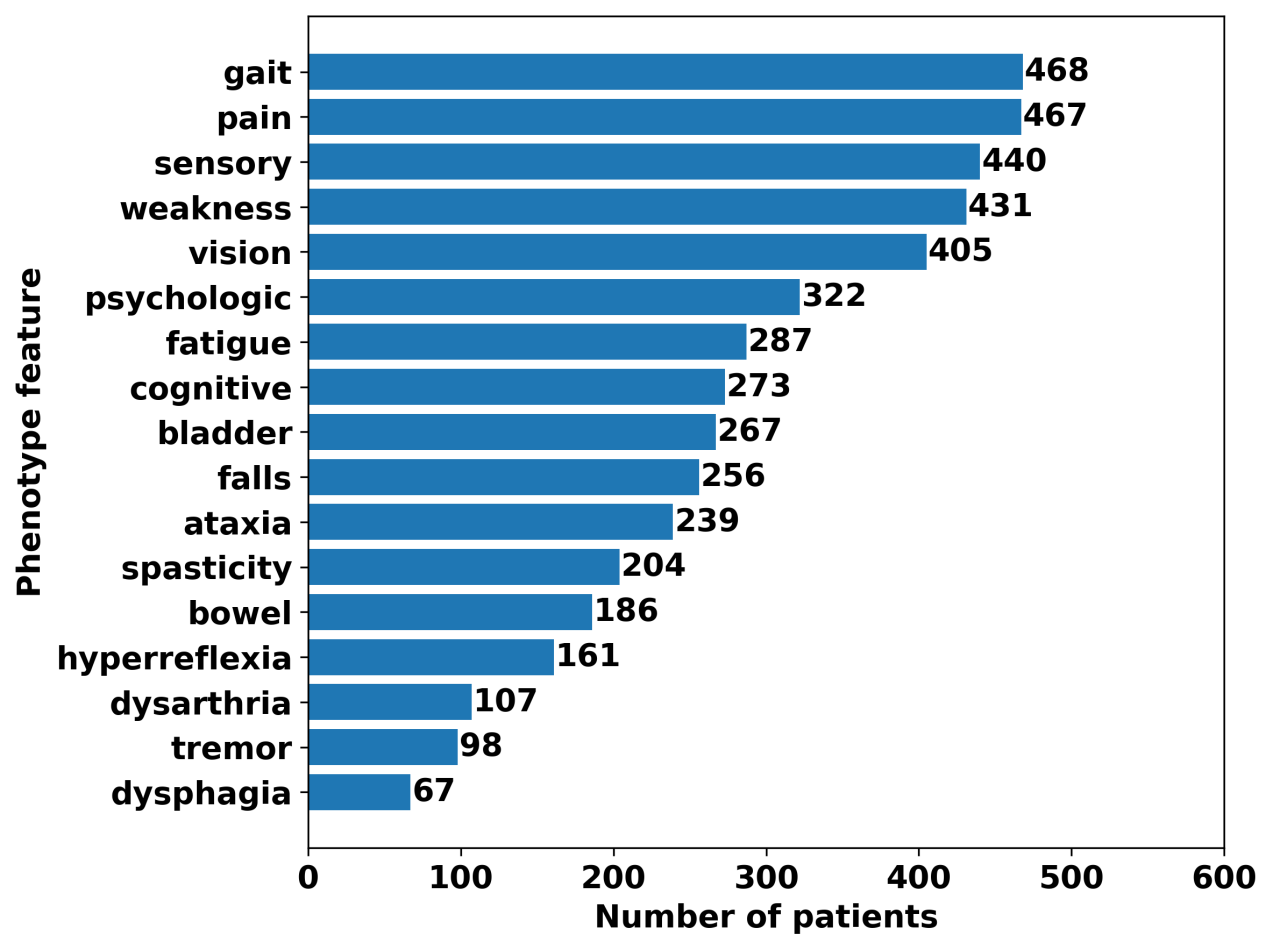
Patient counts by phenotype feature. Most common features were gait disorders, pain, and sensory loss or paresthesias. Counts are across all available notes but each feature is counted only once per patient.

We used a large language model to extract neurological phenotype features from 4617 neurology notes representing 577 patients with multiple sclerosis. Phenotypes were identified and categorized at the whole-note level [16]. Features were aggregated across all available notes for each patient, yielding a single binarized 17-dimensional phenotype vector for each patient. The most common features were abnormal gait, pain, weakness, and abnormal sensation (Figure 2). Patients with no phenotype features were excluded (mean number of features per patient was 8.09 ± 3.35, minimum = 1, maximum = 16, median = 8).

We applied non-negative matrix factorization to the patient phenotype vectors and selected the four-module solution (Figure 1) as the best compromise of interpretability (Table 1) and reconstruction error (Table 3).

The four modules identified by non-negative matrix factorization—sensory-visual-pain, ataxic-spastic-falls, cognitive-psychologic-fatigue, and autonomic-bladder-bowel—were clinically interpretable and corresponded to recognizable patterns of symptom clustering in multiple sclerosis. The highest-weighted features within each module were neurologically coherent and aligned with plausible patterns of dysfunction across pyramidal, cerebellar, sensory, visual, cognitive–affective, and autonomic systems commonly affected in multiple sclerosis.

**Table 1:** Feature loadings for the selected 4-module NMF solution.

Module	Phenotype	Loading
Sensory-visual-pain	sensory	3.172
	pain	3.162
	vision	3.152
	weakness	2.085
	gait	1.931
Ataxic-spastic-falls	ataxia	3.176
	spasticity	2.670
	falls	2.470
	gait	2.077
	weakness	1.716
Cognitive-psychologic-fatigue	psychologic	4.267
	fatigue	3.890
	cognitive	3.594
	sensory	1.475
	pain	0.689
Autonomic-bladder-bowel	bladder	3.548
	bowel	2.748
	vision	1.115
	weakness	1.069
	gait	1.006

Loadings are from the feature matrix of the selected 4-module non-negative matrix factorization solution. Higher loadings indicate stronger contribution of a phenotype feature to the corresponding module.

**Table 2:** Dominant, highly dominant, and archetypal patients by phenotype module type.

Module	Dominant	Highly Dominant	Near Archetypal	Archetypal
Sensory-visual-pain	305	106	58	1
Ataxic-spastic-falls	70	21	2	0
Cognitive-psychologic-fatigue	179	15	7	1
Autonomic-bladder-bowel	24	12	5	0
Total	577	154	72	2

Note. The dominant module was defined as the module with the highest normalized loading. All patients were assigned to one of the four modules. Highly dominant patients had one module accounting for more than 55% of total loading; near-archetypal patients had one module accounting for more than 70% of total loading; archetypal patients had one module accounting for 100% of total loading.

**Table 3:** Relative reconstruction error by number of NMF modules.

Number of Modules (*k*)	Relative Reconstruction Error
2	0.556
3	0.526
4	0.495
5	0.469
6	0.443

Note. Relative reconstruction error was calculated using Equation (1). The k=4 model was selected based on a balance between reconstruction error and clinical interpretability (Figure 1).

**Figure 3:**
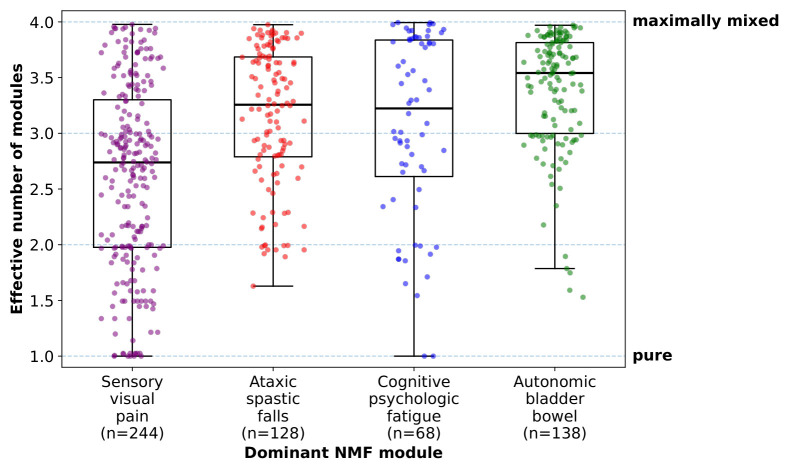
Effective number of phenotype modules by dominant NMF module. Each patient was assigned to the module with the largest normalized NMF weight. The y-axis shows the entropy-derived effective number of modules, which increases with the degree of phenotypic admixture. Values closer to 1 indicate relatively pure, module-dominant profiles, whereas values closer to 4 indicate broader admixture across all four modules. Boxes show the median and interquartile range; whiskers extend to 1.5 times the interquartile range. Individual points represent patients.

The four-module solution allowed each patient to be represented as a normalized four-dimensional compositional vector, with the four module weights summing to 100% (Figure 3).

**Figure 4:**
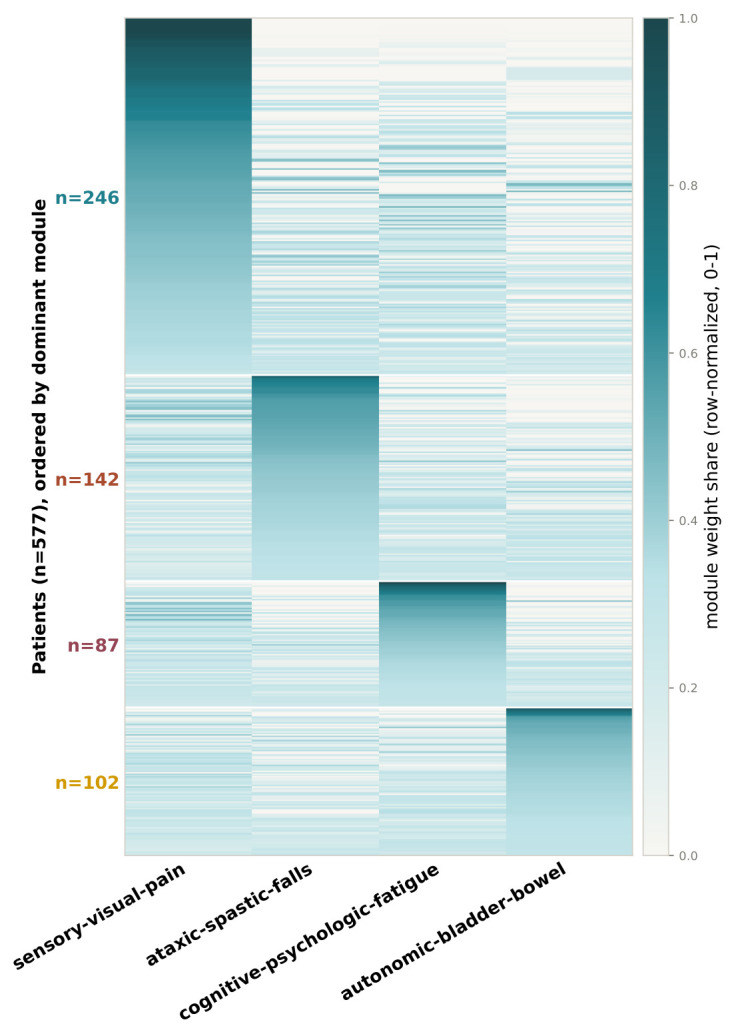
Patient-level NMF module weights, ordered by dominant module. Each row is one patient (*n* = 577); the four columns show the relative weight assigned to each module, with each patient’s weights normalized to sum to 1 (row-normalized) [24]. Patients are grouped into four blocks by their dominant (highest-weight) module—sensory-visual-pain (*n* = 246), ataxic-spastic-falls (*n* = 142), cognitive-psychologic-fatigue (*n* = 87), and autonomic-bladder-bowel (*n* = 102)—and ordered within each block by descending dominant-module weight.

To visualize patient-level module composition directly, we ordered all patients by their dominant module and displayed the row-normalized module weights as a heatmap (Figure 4). The resulting block-diagonal pattern confirms that each module corresponds to a cohesive patient subgroup: within each block the dominant module carries the greatest weight, while the off-diagonal cells indicate the degree of secondary admixture from the remaining modules. The sensory-visual-pain module was the most common dominant phenotype (*n* = 246 of 577, 42.6%), followed by ataxic-spastic-falls (*n* = 142, 24.6%), autonomic-bladder-bowel (*n* = 102, 17.7%), and cognitive-psychologic-fatigue (*n* = 87, 15.1%). Importantly, weight fades gradually across module boundaries rather than transitioning sharply, indicating that patients occupy a continuous spectrum of admixture rather than falling into discrete, mutually exclusive categories.

**Figure 5:**
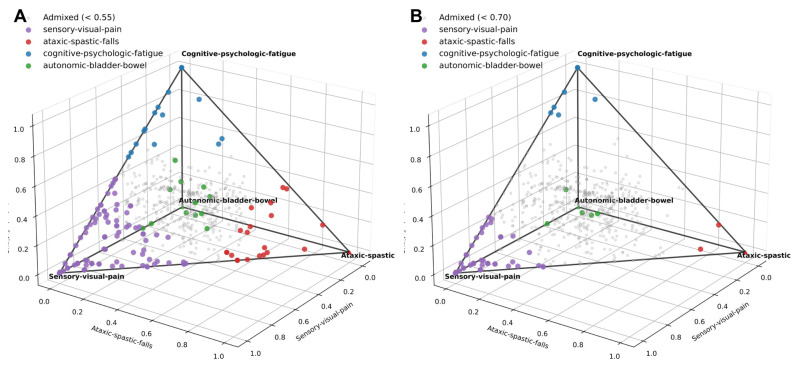
Module weightings for each patient plotted in four-dimensional space, where each vertex represents a relatively pure presentation of one module phenotype. (**Panel A**) shows patients colored by a dominant module when the dominant module contributed >0.55 of the total phenotype loading. (**Panel B**) shows patients colored by a dominant module when the dominant module contributed >0.70 of the total phenotype loading. Patients shown in gray have admixed module phenotypes.

**Figure 6:**
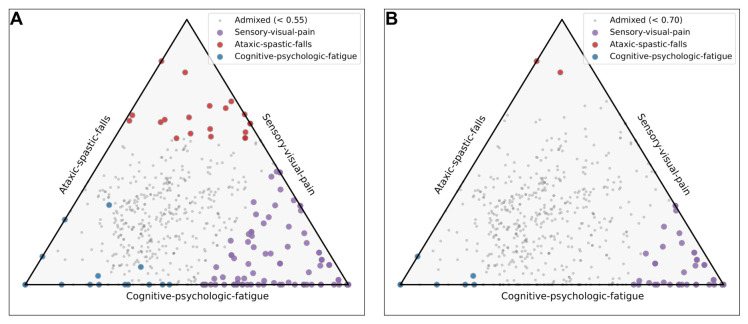
Simplex view of the tetrahedron showing three of the four vertices. Patients closer to a vertex have purer module phenotypes. The vertices shown represent the ataxic-spastic-falls (red), sensory-visual-pain (purple), and cognitive-psychologic-fatigue (blue) modules. (**Panel A**) shows patients with >0.55 loading on a single module; (**Panel B**) shows patients with >0.70 loading on a single module. Markers are colored according to the dominant module.

**Figure 7:**
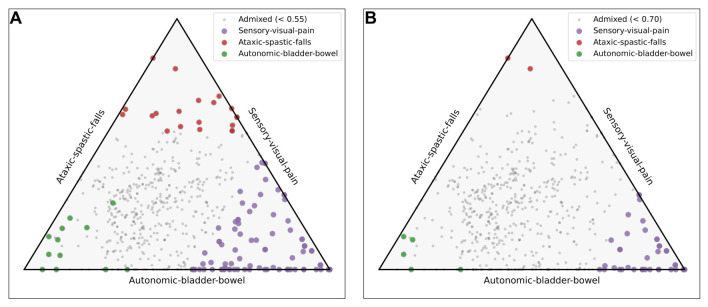
Simplex view of the tetrahedron showing three of the four vertices. Patients closer to a vertex have purer module phenotypes. The vertices shown represent the ataxic-spastic-falls (red), sensory-visual-pain (purple), and autonomic-bladder-bowel (green) modules. (**Panel A**) shows patients with >0.55 loading on a single module; (**Panel B**) shows patients with >0.70 loading on a single module. Markers are colored according to the dominant module.

Because all module weights were non-negative and row-normalized to sum to 100%, each patient could be visualized within a regular tetrahedral space, or 3-simplex, in which each vertex represented a pure archetype corresponding to one of the four modules. Patients mapped near a vertex in the tetrahedral projection (Figure 5) can be interpreted as relatively pure examples of a single module (near-archetypes), whereas patients located in the interior represent admixtures of multiple modules. Patients positioned along edges can be interpreted as mixtures of two modules, whereas patients located on faces can be interpreted as mixtures of three modules. To highlight degrees of dominance, patient markers in the tetrahedral projection were identified according to two thresholds: patients were classified as highly dominant if one module accounted for more than 55% of the total loading and as near-archetypal if one module accounted for more than 70% of the total loading [20,21,22,23]. The tetrahedron should be interpreted as a convenient visualization of normalized four-module composition rather than as independent evidence for discrete patient clusters (Figure 5). Individual faces of the tetrahedron can be visualized as triangular simplices, in which each vertex represents the pure form of one of the four additive modules (Figure 6 and Figure 7).

**Figure 8:**
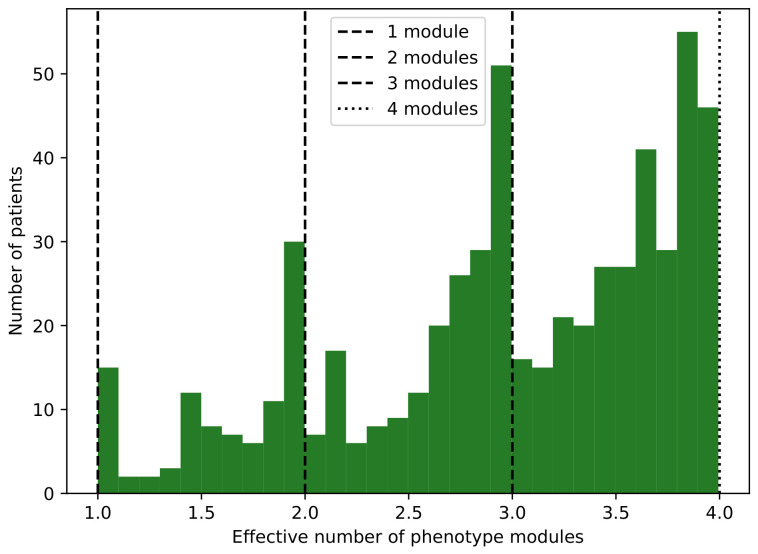
Histogram of the effective number of phenotype modules. The effective number of modules quantifies the degree of admixture among the four phenotype modules for each patient. It is derived from Shannon entropy and ranges from 1.0, indicating a highly pure phenotype assignment, to 4.0, indicating maximal admixture. Most patients had an effective number of modules greater than 2.5 and were considered moderately to highly admixed.

As a second measure of phenotypic admixture, we calculated the Shannon entropy of the four normalized module weights for each patient. When 100% of the loading is concentrated in a single module, entropy is 0; when the loadings are distributed equally across all four modules, entropy reaches its maximum value of ln(4)=1.386. Entropy values were converted to the effective number of modules using the formula $e^{H}$, where *H* is the Shannon entropy (Figure 8). The effective number of modules therefore ranges from 1 to 4, with values near 1 indicating that a patient’s phenotype is concentrated within a single module and values near 4 indicating maximal admixture across all four modules. Importantly, a patient may have multiple phenotypic features within the same module group, such as sensory symptoms, pain, and visual symptoms, while still having an effective module count near 1 if those features map predominantly to a single latent module.

## 4. Discussion

In this study, we modeled the phenotype of multiple sclerosis as a non-negative mixture of additive signs and symptoms modules derived from empirical clinical-note data. The motivation for this approach is grounded in the long-recognized polysymptomatic nature of MS [2]. By the mid-twentieth century, MS was defined as an autoimmune demyelinating disease characterized by lesions disseminated in time and space [25]. Kurtzke’s Expanded Disability Status Scale (EDSS) and Functional Systems Scores (FSS) [9,10,26] codified this clinical heterogeneity by distributing disability across pyramidal, cerebellar, brainstem, sensory, bowel and bladder, visual, and cerebral systems [10]. Although the FSS provide a clinically intuitive multi-axis framework, it assigns signs and symptoms to functional systems using clinician-defined categories. In contrast, the modules identified here were derived from data-driven co-occurrence patterns among note-derived phenotype features.

This distinction is important because MS phenotype is not stereotyped. Individual patients accumulate different combinations of sensory, motor, cognitive, and autonomic impairments over time, reflecting demyelinating lesions distributed across different CNS locations and disease episodes.

The empirical feature distributions in this cohort supported this view. Most patients had multiple phenotype features documented across their available notes, and the most frequent features included gait impairment, pain, weakness, and sensory symptoms; Figure 2. Thus, the input data did not consist of isolated single-symptom presentations, but of broad patient-level phenotype profiles accumulated over time. This polysymptomatic structure provided the rationale for modeling MS phenotype as a mixture of latent modules rather than as a set of mutually exclusive clinical categories.

The observed phenotype can be viewed as the additive superposition of system-specific impairments rather than as movement along a single severity axis. Under this interpretation, NMF provides a natural framework for representing each patient as a non-negative mixture of latent symptom modules. We examined two-, three-, four-, and five-module solutions and selected the four-module solution as the preferred model because it provided the clearest clinical interpretation while maintaining reasonable reconstruction error. This choice should be viewed as a balance between model fit and interpretability rather than as evidence for a uniquely optimal number of biological subtypes.

The four-module solution identified clinically coherent modules corresponding to sensory-visual-pain, ataxic-spastic-falls, cognitive-psychologic-fatigue, and autonomic-bladder-bowel patterns. These empirically derived modules broadly resembled familiar neurological functional domains, including sensory/visual pathways, pyramidal–cerebellar motor systems, distributed cognitive–affective networks, and autonomic/spinal cord pathways with supraspinal modulation.

When the four non-negative module weights were normalized to sum to 100%, patients could be visualized within a tetrahedral, or 3-simplex, space [24]. This visualization provides an intuitive geometry for module composition: vertices represent pure module profiles, edges represent two-module mixtures, faces represent three-module mixtures, and interior points represent admixtures of all four modules. The tetrahedron should be interpreted as a convenient visualization of normalized four-module composition rather than as independent evidence for discrete patient clusters. In this study, most patients occupied admixed regions of the phenotype space, although a subset showed relatively pure or strongly dominant patterns. These purer phenotypes were most often sensory-visual-pain dominant, whereas relatively pure ataxic-spastic-falls, cognitive-psychologic-fatigue, and autonomic-bladder-bowel phenotypes were less common. Only two patients occupied true vertices of the tetrahedron, corresponding to pure sensory-visual-pain and pure cognitive-psychologic-fatigue profiles (Table A3).

The presence of relatively pure vertex-adjacent phenotypes invites comparison with archetypal analysis, a method designed to identify extreme representative profiles on the convex hull of the data [24]. Archetypal approaches have been used to define clinically interpretable extreme disease states from longitudinal clinical data. For example, Trescato et al. [27] derived archetypal phenotypes in amyotrophic lateral sclerosis and used them to model disease-progression trajectories, illustrating the value of low-dimensional phenotype representations for studying clinical heterogeneity. The present analysis differs in an important respect: whereas archetypal analysis identifies extreme representative disease states, non-negative matrix factorization identifies additive latent phenotype modules that may coexist within the same patient. Thus, the vertices of the tetrahedron should not be interpreted as archetypes discovered by archetypal analysis, but rather as limiting cases of normalized four-module composition.

The predominance of admixed phenotypes is consistent with clinical experience: as MS evolves, patients often accumulate deficits across multiple functional systems rather than remaining confined to a single domain. At the same time, the existence of relatively module-pure patients suggests that some individuals may have phenotypes dominated by particular functional systems. Whether these module-dominant and archetypal patterns reflect biologically meaningful subtypes, stochastic lesion distribution, differences in disease stage, or differences in documentation remains uncertain. If module-dominant groups show distinct MRI lesion topography, regional atrophy, immunologic signatures, fluid biomarkers, genetic risk profiles, or treatment responses, this would support the biological relevance of the module structure. Conversely, if biological markers are similar across module-dominant groups, the modules may primarily reflect the geometry of CNS functional organization under a common pathogenic process.

The principal practical value of this approach is that it converts heterogeneous clinical-note phenotypes into quantitative patient-level module scores. These scores have three components: (1) the identity of the dominant symptom module, (2) the magnitude of the dominant module, and (3) the effective number of modules. These scores may serve as predictors, outcomes, or stratification variables in future models of relapse risk, disability progression, treatment response, MRI lesion distribution, regional atrophy, or biomarker profiles.

Conventional one-dimensional severity scales do not explicitly capture both module dominance and degree of admixture. Even multi-axis clinical instruments such as the Kurtzke Functional Systems Scores provide domain-specific scores but do not directly yield a normalized measure of phenotypic admixture such as the effective number of modules (Figure 8). In this sense, the four-module representation has a regularization-like effect: it trades some feature-level granularity for a more stable and interpretable description of recurring phenotypic patterns.

These findings do not establish distinct MS disease entities. Rather, they provide a quantitative framework for representing MS phenotypic diversity and for generating testable hypotheses about clinically or biologically meaningful subgroups. Future studies should determine whether these NMF-derived module scores are reproducible across cohorts and whether they predict independent measures of disease activity, progression, treatment response, or biological mechanism.

### 4.1. Relation to Prior MS Phenotype Studies

Prior MS phenotyping studies can be broadly divided into two related approaches (Table 4). Some studies use symptom features to assign patients to discrete classes or clusters. Others reduce correlated symptom features into a smaller number of latent dimensions, factors, or communities. Both approaches begin with a patient-by-feature matrix, but they differ in whether the primary object of analysis is the patient or the feature structure.

Others have examined the problem of categorizing MS subjects by phenotype (Table 4). Most studies begin with a rectangular data matrix of *m patients* (rows) and *n phenotype features* (columns). In general, two different but complementary approaches have been taken to these *m x n* data matrices. One approach is to use the feature columns to group or cluster like patients into a small number of interpretable clusters or classes. The other approach has been to group or factorize the features into a smaller number of interpretable factors.

Shahrbanian et al. [28] used hierarchical clustering to examine relationships among nine MS symptom variables and identified three broad variable clusters: cognitive-emotional symptoms, pain-fatigue-sleep symptoms, and spasticity-balance symptoms. Although clinically intuitive, this analysis produced a relatively coarse symptom grouping rather than a patient-level compositional phenotype model. Gulick [29] used factor analysis to reduce 22 phenotypic features to 5 factors: skeletal-motor (weakness, ataxia, spasticity), elimination (bowel and bladder), emotional (depression and anxiety), sensory, and brainstem/visual.

Ajdacic-Gross et al. [30] examined 20 symptom phenotypes in 1942 multiple sclerosis subjects and derived six distinct classes through latent class analysis, which included multiple symptoms (14.1%), gait-balance (13.5%), fatigue-weakness (11.7%), gait-paralysis (23.9%), vision (21.3%), and paresthesia (15.4%). De Nadai et al. applied latent profile analysis to 11 Multiple Sclerosis Patient-Reported Outcome (MS-PRO) scales, which captured patient-reported impairment across mobility, hand function, vision, fatigue, cognition, bladder/bowel function, sensory symptoms, spasticity, pain, depression, and tremor/coordination domains. They derived nine distinct subtypes including normal functioning, fatigue, sensory, somatic, somatic plus cognitive, severe disability, poor mobility, moderate disability, and physical symptoms. Howlett-Prieto et al. [32] applied the Louvain algorithm to detect network communities in a 113 patient by 17 symptom feature array and found five unipartite communities (pain, fatigue, cognitive, sensory, and gait-weakness-hypertonia).

Taken together, these studies support the view that MS phenotype is multidimensional and cannot be adequately represented by a single severity axis. However, they differ from the present study in three important respects. First, several prior studies used patient-reported outcomes or symptom-onset questionnaires, whereas the present analysis used phenotype features extracted from longitudinal clinical notes. Second, clustering, latent class analysis, and latent profile analysis assign patients to discrete groups, whereas NMF represents each patient as a mixture of additive symptom modules. Third, prior cluster or class solutions generally produced mutually exclusive patient categories, while the present approach explicitly quantifies admixture through normalized module weights, module dominance, entropy, and the effective number of modules. Thus, the present findings should be viewed not as a replacement for prior MS phenotyping studies, but as a complementary representation of MS phenotype as a continuous, modular, and compositional space.

### 4.2. Limitations and Future Directions

This study has several limitations that also define important directions for future work. First, phenotyping was performed by a large language model rather than by manual expert review, although prior work has shown near-human-level performance of the same model on related neurology phenotyping tasks [16]. Future studies should include additional clinician validation of the automated phenotyping approach, ideally using independent note samples and multiple expert reviewers. Because independent clinician adjudication was not repeated on the full 4617-note corpus, residual annotation error may propagate into the module structure; additionally, the requirement for complete physician notes may select toward encounters with more thorough documentation.

Second, all notes were drawn from a single urban academic safety-net medical center. As a result, the cohort may not be representative of MS populations seen in other clinical settings, particularly with respect to disease severity, disability burden, socioeconomic factors, access to care, and documentation practices. We also did not account for potential differences in documentation style across physicians, clinics, or health systems. The study lacked an independent internal or external validation cohort and should therefore be considered proof-of-concept rather than definitive. Future work should test whether the same four-module structure is reproducible in additional MS cohorts and across institutions.

Third, phenotype features were aggregated across all available notes for each patient to reduce variability in single-note documentation, but we did not explicitly model the number or timing of notes, nor did we examine the temporal evolution of module weights. Longitudinal analyses could determine whether module scores change over time with relapse, remission, progression, or disease-modifying therapy, and whether shifts in module composition provide clinically useful information beyond static phenotype burden.

In addition, phenotype features were scored on a binary basis as absent or present. We did not attempt to grade severity, frequency, laterality, anatomical distribution, or functional impact. As a result, a mild symptom and a severe symptom could contribute equally to the feature matrix. The feature set was intentionally limited to commonly documented MS phenotypes, and some less common manifestations, such as tinnitus, hearing loss, seizures, or other episodic symptoms, were not included. Future studies could extend this framework by incorporating severity grading, anatomical localization, temporal dynamics, and a broader set of MS-related features. As a future enhancement, phenotype features could be mapped to concepts in standard ontologies such as the Human Phenotype Ontology (HPO) or SNOMED CT.

Fourth, we focused on non-negative matrix factorization (NMF) and did not formally compare the four-module solution with alternative dimensionality-reduction, matrix-factorization, or clustering methods, such as principal components analysis, independent components analysis, factor analysis, latent class analysis, latent Dirichlet allocation/topic modeling, archetypal analysis, hierarchical or consensus clustering, Gaussian mixture models, biclustering, or graph/community-detection approaches. NMF was selected because its non-negative, parts-based representation is well-suited to modeling additive symptom burden and produces clinically interpretable modules. However, future studies should determine whether similar phenotype structure is recovered using alternative methods and should compare solutions using stability, reconstruction error, interpretability, and external clinical validity. In exploratory analyses we applied independent components analysis and found that the resulting mixed-sign components were not interpretable as additive symptom modules, supporting the choice of NMF for this non-negative, parts-based representation. A formal head-to-head comparison of unsupervised methods on the same data is identified as future work.

We also did not attempt to reconstruct Kurtzke Extended Disability Status Scale (EDSS) or Functional Systems Scores (FSS) from the available notes [9,10,26,34]. The notes analyzed in this study were routine clinical-care documents rather than standardized research or clinical-trial assessments, and they did not consistently contain the graded severity, functional-system scoring, ambulation distance, or assistance-level information required for valid EDSS or FSS assignment. Future studies linking note-derived module scores to prospectively collected or carefully curated EDSS and FSS measures could determine whether module dominance and phenotypic admixture provide information complementary to established MS disability scales.

Furthermore, phenotype extraction was dependent on the content and quality of clinical documentation. Physicians vary in documentation style, completeness, and attention to neurological detail, and these differences may influence the observed phenotype matrix independently of the patient’s true clinical state. Thus, some variation in phenotype burden may reflect documentation practices rather than biological or clinical differences among patients. Although physician-level documentation quality could potentially be modeled using indirect features such as note length, structure, vocabulary diversity, frequency of negative findings, or evidence of templated text, such an analysis was outside the scope of the present study.

A central limitation of note-derived phenotyping is that absence of documentation does not imply absence of symptoms; clinical notes are not standardized symptom inventories. This concern is supported empirically by the aggregation-window sensitivity analysis. When phenotype burden was reconstructed from a single note (the first or last available), feature prevalence was substantially lower than in the all-notes cumulative aggregate, and documentation consistency varied by domain, with motor and sensory findings recorded more reliably than fatigue, cognition, and mood. The autonomic-bladder-bowel module was markedly less stable when derived from single notes (matched loading correlation 0.06 from the first note and 0.22 from the last note, versus 0.99 or higher in the all-notes analysis), consistent with intermittent documentation of bowel and bladder symptoms. These findings support the use of cumulative aggregation across all available notes and reinforce that the modules represent documentation-derived symptom dimensions rather than complete symptom inventories. The institution- and template-specific nature of documentation is itself a determinant of the observed module structure: EHR templates structure the domains prompted at each encounter, and clinicians selectively update rather than re-document established problems [17,18]. Local demographics, care access, and these institutional documentation patterns may therefore influence the derived modules, and external replication in an independent cohort is a priority for future work. The need for more structured and templated MS documentation has been recognized [35,36]: in a survey of neurology trainees across dedicated MS outpatient clinics, records were frequently judged too short with relevant data missing, and respondents called for structured records with closed fields and disease timelines [19].

Finally, we did not correlate module scores with independent markers of MS disease activity or progression, such as MRI lesion burden, regional atrophy, relapse rate, disability progression, serum or CSF biomarkers such as neurofilament light chain or GFAP, or genomic, immunologic, or methylomic data. Integrating NMF-derived phenotypic modules with these biological and clinical measures will be necessary to determine whether module-dominant or highly admixed phenotypes correspond to biologically meaningful MS subtypes or clinically useful prognostic groups. Future work should assess whether these modules can predict relapse, disability progression, or treatment responses or whether these modules correlate with MRI, biomarker, or genetic data [1].

## 5. Conclusions

Multiple sclerosis lacks a single invariant phenotypic core and may be better described as a superposition of impairments across multiple functional systems. Using non-negative matrix factorization of routinely collected neurology notes, we found that MS phenotypes can be parsimoniously represented as mixtures of four latent symptom modules: sensory-visual-pain, ataxic-spastic-falls, cognitive-psychologic-fatigue, and autonomic-bladder-bowel. This representation places each patient within a two-axis interpretive framework: the dominant module identifies the patient’s predominant phenotype direction, whereas the effective number of modules quantifies the degree of phenotypic admixture across modules (Figure 8). Most patients showed admixed phenotypes, but a substantial minority had relatively pure or strongly dominant module patterns. These results provide a simple, interpretable framework for quantifying phenotypic diversity in MS and generate module-level descriptors that may be useful in downstream analyses of disease progression, MRI burden, treatment response, biomarkers, and documentation-derived symptom modules whose biological relevance remains to be established. The modules derived here represent documentation-derived symptom dimensions rather than biological disease entities, and their correspondence to underlying pathophysiology will require validation against imaging, biomarker, and longitudinal data in independent cohorts.

## Figures and Tables

**Table 4:** Selected prior data-driven studies of multiple sclerosis phenotype structure.

Study	Data/Features	Method	Main Finding
Shahrbanian et al. [28]	9 MS symptom variables	Hierarchical clustering	Identified three broad symptom-variable clusters: cognitive-emotional, pain-fatigue-sleep, and spasticity-balance.
Gulick [29]	22 phenotypic features	Factor analysis	Reduced symptoms to five factors: skeletal-motor, elimination, emotional, sensory, and brainstem/visual.
Ajdacic-Gross et al. [30]	20 onset-symptom phenotypes by 1942 MS subjects	Latent class analysis	Identified six onset-symptom classes, including multiple-symptom, gait-balance, fatigue-weakness, gait-paralysis, vision, and paresthesia classes.
De Nadai et al. [31]	11 MS-PRO scales by 6619 MS subjects	Latent profile analysis	Identified nine patient-reported impairment profiles, largely organized by low, moderate, and high mobility impairment.
Howlett-Prieto et al. [32]	113 patients by 17 symptom features	Louvain network community detection	Identified five symptom communities: pain, fatigue, cognitive, sensory, and gait-weakness-hypertonia.
Eshaghi et al. [33]	MRI-derived measures by 6322 MS patients (3068 validation)	SuStaIn (subtype and staging inference)	Identified three MRI-based subtypes—cortex-led, normal-appearing white matter-led, and lesion-led; the lesion-led subtype predicted disability progression and relapse.
Present study	577 patients by 17 note-derived phenotype features	Non-negative matrix factorization	Identified four additive symptom modules and represented each patient as a normalized mixture of module weights.

## Data Availability

Python code and data is available on request.

## Appendix A

**Table A1:** Variables included in the analytic data file.

Variable	Type	Description
*Metadata*		
note_id	string	Unique note identifier
patient_id	string	Unique patient identifier
note_count	integer	Number of notes available for the patient
note_num	integer	Sequential note number for the patient
note_date	date	Date of clinical note
elapsed_days	integer	Days elapsed from first available note
clinic	categorical	Clinic or practice setting
note_type	categorical	Type of clinical note
dx_codes	string	ICD-10-CM diagnosis codes
age	integer	Age in years
gender	categorical	Male or female
race	categorical	White, Black, or Other
ethnicity	categorical	Hispanic or non-Hispanic
note_text	string	Text of EHR note
*Phenotype features*		
dysarthria	binary	Dysarthria or slurred speech
dysphagia	binary	Difficulty swallowing
spasticity	binary	Spasticity or hypertonia
hyperreflexia	binary	Hyperreflexia, clonus, or increased reflexes
vision	binary	Visual, optic nerve, nystagmus, or eye movement symptoms
weakness	binary	Any weakness or loss of strength
sensory	binary	Sensory loss or paresthesia
ataxia	binary	Ataxia or incoordination
tremor	binary	Tremor
*Phenotype features*		
bladder	binary	Urinary incontinence, urgency, or retention
bowel	binary	Bowel incontinence or constipation
cognitive	binary	Any cognitive symptom
falls	binary	Falling or recurrent falls
fatigue	binary	Tiredness or fatigue or asthenia
gait	binary	Gait impairment or balance difficulty
pain	binary	Any pain or burning sensation
*Derived module metrics*		
sensory_visual_pain_loading	float	
ataxia_spasticity_falls_loading	float	
cognitive_psychological_fatigue_loading	float	
autonomic_bladder_bowel_loading	float	
dominant_module	categorical	Module with the highest contribution
effective_modules	float	Effective number of phenotype modules

**Table A2:** Stability of the four-module NMF solution under repeated 90% subsampling.

Module	Median *r*	Q1	Q3	Min.	Max.
Autonomic-bladder-bowel	0.995	0.992	0.997	0.973	0.999
Cognitive-psychologic-fatigue	0.997	0.995	0.998	0.987	1.000
Ataxic-spastic-falls	0.997	0.995	0.998	0.987	0.999
Sensory-visual-pain	0.999	0.998	0.999	0.996	1.000

Note. Stability was assessed using 100 repeated 90% subsampling runs: in each run a random 90% sample of patients was drawn, the four-module NMF was refit, and each subsample module was matched to its full-cohort counterpart. Stability is reported as the Pearson correlation between the 17-dimensional feature-loading vectors of each matched module pair. Across the four modules, median matched correlations ranged from 0.995 to 0.999, confirming that the four-module solution is stable under subsampling.

**Table A3:** Empirical occupancy of tetrahedral phenotype vertices.

Case	Module	Max Weight	Second Weight	Vertex	Interpretation
1	cognitive-psychologic-fatigue	1.00	None	Yes	Pure anchor
2	Sensory-visual-pain	1.00	None	Yes	Pure anchor
3	Autonomic-bladder-bowel	0.89	Sensory-visual-pain (0.06)	No	Near-pure boundary
4	Ataxic-spastic-falls	0.84	Cognitive-psychologic-fatigue (0.14)	No	Near-pure boundary

Note. A pure vertex was defined as a patient with normalized module weight equal to 1.00. Although the four-module NMF solution defines an ideal tetrahedral phenotype space, the observed MS cohort did not fully occupy this space. Pure empirical anchors were observed only for the cognitive-psychologic-fatigue and sensory-visual-pain modules. Case IDs are arbitrary, but each exemplar case is chosen from the full set of 577 multiple sclerosis patients.
